# Community impacts of anthropogenic disturbance: natural enemies exploit multiple routes in pursuit of invading herbivore hosts

**DOI:** 10.1186/1471-2148-10-322

**Published:** 2010-10-23

**Authors:** James A Nicholls, Pablo Fuentes-Utrilla, Alexander Hayward, George Melika, György Csóka, José-Luis Nieves-Aldrey, Juli Pujade-Villar, Majid Tavakoli, Karsten Schönrogge, Graham N Stone

**Affiliations:** 1 Institute of Evolutionary Biology, University of Edinburgh, Ashworth Labs, King's Buildings, Edinburgh EH9 3JT, UK; 2 Department of Zoology, University of Oxford, South Parks Road, Oxford OX1 3PS, UK; 3 Pest Diagnostic Laboratory, Plant Protection & Soil Conservation Directorate of County Vas, Ambrozy setany 2, 9762 Tanakajd, Hungary; 4 Hungarian Forest Research Institute, Mátrafüred Research Station, 3232 Mátrafüred, Hungary; 5 Departamento de Biodiversidad y Biología Evolutiva, Museo Nacional de Ciencias Naturales (CSIC), c/José Gutiérrez Abascal, 2. Madrid. E-28006, Spain; 6 Universitat de Barcelona, Facultat de Biologia, Departament de Biologia Animal, Avda. Diagonal 645, ES-08028, Barcelona, Spain; 7 Lorestan Agricultural and Natural Resources Research Center, Khorramabad, Lorestan, P.O. Box 348, Iran; 8 CEH Wallingford, Maclean Building, Benson Lane, Crowmarsh Gifford, Wallingford, Oxfordshire, OX10 8BB, UK

## Abstract

**Background:**

Biological invasions provide a window on the process of community assembly. In particular, tracking natural enemy recruitment to invading hosts can reveal the relative roles of co-evolution (including local adaptation) and ecological sorting. We use molecular data to examine colonisation of northern Europe by the parasitoid *Megastigmus stigmatizans *following invasions of its herbivorous oak gallwasp hosts from the Balkans. Local host adaptation predicts that invading gallwasp populations will have been tracked primarily by sympatric Balkan populations of *M. stigmatizans *(Host Pursuit Hypothesis). Alternatively, ecological sorting allows parasitoid recruitment from geographically distinct populations with no recent experience of the invading hosts (Host Shift Hypothesis). Finally, we test for long-term persistence of parasitoids introduced via human trade of their hosts' galls (Introduction Hypothesis).

**Results:**

Polymorphism diagnostic of different southern refugial regions was present in both mitochondrial and nuclear microsatellite markers, allowing us to identify the origins of northern European invaded range *M. stigmatizans *populations. As with their hosts, some invaded range populations showed genetic variation diagnostic of Balkan sources, supporting the Host Pursuit Hypothesis. In contrast, other invading populations had an Iberian origin, unlike their hosts in northern Europe, supporting the Host Shift Hypothesis. Finally, both British and Italian *M. stigmatizans *populations show signatures compatible with the Introduction Hypothesis from eastern Mediterranean sources.

**Conclusions:**

These data reveal the continental scale of multi-trophic impacts of anthropogenic disturbance and highlight the fact that herbivores and their natural enemies may face very different constraints on range expansion. The ability of natural enemies to exploit ecologically-similar hosts with which they have had no historical association supports a major role for ecological sorting processes in the recent assembly of these communities. The multitude of origins of invading natural enemy populations in this study emphasises the diversity of mechanisms requiring consideration when predicting consequences of other biological invasions or biological control introductions.

## Background

Growing numbers of natural communities are being disrupted by biological invasions resulting from human activity. The ecological impacts of these invasions depend in part on whether they facilitate knock-on invasions by other taxa, such as natural enemies, and the prevalence of interactions between these secondary invaders and native taxa. The scale and speed of natural enemy range expansion and the likelihood of shifts to non-target hosts are particularly crucial when predicting community impacts of intentionally released biological control agents [1]. Given the complexity of most ecological systems [2], predicting such impacts is extremely challenging. More generally, monitoring how communities develop around invading species can allow testing of alternative models of community assembly and evolution [3]. Here we use human-facilitated range expansion affecting three trophic levels in a plant-herbivore-natural enemy system to assess the relative importance of long-term historical associations versus ecological sorting [4-6].

**Figure 1:**
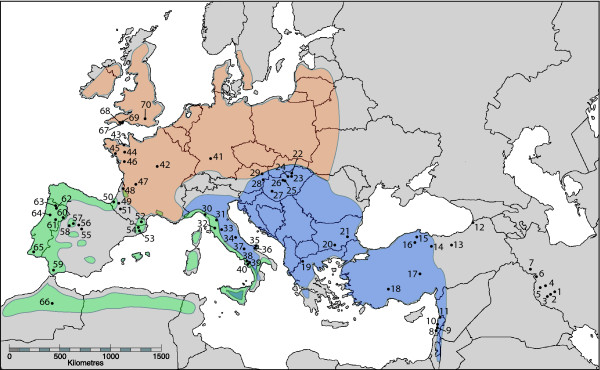
**Map indicating *Megastigmus stigmatizans *sampling locations and the major European *Quercus *section Cerris oak distributions**. Green shading indicates the native distribution of *Q. suber*, blue the native distribution of *Q. cerris*, and orange the introduced distribution of *Q. cerris*. Sites are numbered as in Additional file 1.

Our tri-trophic study system comprises gall-inducing herbivorous wasps (Hymenoptera: Cynipidae), whose distributions within the Western Palaearctic are tightly coupled to those of their host oak trees, genus *Quercus *[7], and parasitoid natural enemies [8]. Species in one major clade of *Andricus *gallwasps have lifecycles involving host alternation between two oak lineages - the black oaks of *Quercus *section Cerris (e.g. *Q. cerris *and *Q. suber*) and the white oaks of *Quercus *section Quercus (e.g. *Q. robur *and *Q. petraea*). In the Western Palaearctic natural co-existence of these two oak sections (and hence host-alternating gallwasps) is restricted to southern Europe, Asia Minor and the Middle East; only section Quercus oaks are native to Europe north of the Pyrenees, Alps and Carpathians [9,10]. However, over the last 400 years the region in which both oak sections grow together has been dramatically expanded by widespread human planting in northern and western Europe of Turkey oak *Q. cerris *(Figure 1), a section Cerris species native to central and eastern Europe and Asia Minor. This introduction has triggered multiple invasions by a suite of host-alternating gallwasps, reaching northwards to Scotland and westwards to the northern slopes of the Pyrenees [8,9,11]. Genetic data show that all invading gallwasp populations have originated from within the native Balkan range of *Q. cerris*, with no range expansion by Iberian peninsula populations [9,10,12]. In Iberia, cork oak (*Q. suber*) replaces *Q. cerris*, and Iberian gallwasp populations have proven unable to make the host switch from *Q. suber *to *Q. cerris *that is necessary for northwards range expansion.

This study concerns the response of the third trophic level to gallwasp range expansion. Oak gallwasps support rich communities of parasitoid natural enemies [8,13], and gallwasp range expansions triggered by planting of *Q. cerris *represent continental-scale experiments in the assembly of such communities. Parasitoids have recruited onto range-expanding gallwasps in invaded regions [11,13,14], but their origins remain unclear. However, determining where these parasitoids come from can reveal underlying mechanisms in gallwasp community assembly. If shared spatial histories and co-evolution (including local adaptation) underpin species interactions [5,15], we expect invading gallwasps to be pursued by enemies from the same Balkan origin (the **Host Pursuit Hypothesis**); this hypothesis predicts no parasitoid recruitment from alternative populations (for example in Iberia) that have had no shared spatial or co-refugial history with the invading hosts. An alternative model emphasises community assembly by ecological sorting, with a reduced impact of co-evolution or local adaptation [4,6]. This model allows exploitation of invading hosts by parasitoid populations that have no recent history of exposure to invaders but that attack ecologically similar hosts (the **Host Shift Hypothesis**). More specifically, whilst this hypothesis does not preclude some degree of host pursuit from the Balkans, it additionally predicts that expanding parasitoid populations could escape from Iberia even though their gallwasp hosts have not.

A third scenario potentially applies to this gallwasp system, involving co-introduction of both gallwasps and their parasitoid enemies (the **Introduction Hypothesis**). Accidental co-introduction is a feature of community development around invading hosts [16] and is possible because galls (and unintentionally their occupants) have long been traded as a source of tannins for the manufacture of inks and dyes [[9] and references therein]. The source for such galls was the eastern Mediterranean (particularly Lebanon and south-eastern Turkey), with Roman Italy as the major historical destination. More recently, vast numbers of galls of the host-alternator *Andricus kollari *were imported into south-western Britain from the eastern Mediterranean during the 1840s [9]. *Andricus kollari *rapidly colonised Britain, and genetic data show these introduced populations were derived from at least two eastern Mediterranean sources [9]. It is likely that parasitoids also escaped from these imported galls, but it remains unknown whether any populations successfully established. However, if they did establish we would expect British and Italian populations (but not other European populations) to exhibit genetic variation diagnostic of the eastern Mediterranean.

These three hypotheses can be discriminated using population genetic data for parasitoids specialising on host-alternating gallwasps, whose populations in northern Europe therefore must also be invading or introduced. Our approach is to use a combination of mitochondrial sequence and nuclear microsatellite data to identify genetic variation diagnostic of southern and eastern regions relevant to our hypotheses. Screening of invaded range populations then allows assessment of the support for the Host Pursuit, Host Shift and Introduction Hypotheses (genetic variation shared with the Balkans, Iberia or eastern Mediterranean, respectively). The use of multiple nuclear markers plus the independently evolving mitochondrial genome also allows further examination of the histories of range expanding populations, such as the extent to which invading populations from different origins maintain their genetic distinctiveness, or the role of sex-biased dispersal [17,18].

Here we apply this approach to the parasitoid *Megastigmus stigmatizans *(Chalcidoidea, Torymidae). This species is native to southern Europe (including Iberia), Asia Minor and the Middle East where it attacks predominantly both widespread and refuge-specific host-alternating gallwasp species, and also now attacks invading gallwasp populations throughout northern Europe (see Table 1) [14,19]. Additionally, we target this species as information from it is relevant to ongoing work on a related Asian torymid parasitoid, *Torymus sinensis*, recently released in Italy in an attempt to control an introduced and invading gallwasp pest of chestnuts [20,21]. Native oak gall parasitoids have been reared from this invading gallwasp, indicating that oak and chestnut gallwasp communities are trophically linked [20]. We know of no study to date of the potential impacts of this release on native Western Palaearctic gallwasp communities, so patterns shown by *M. stigmatizans *may reveal possible outcomes.

**Table 1:** Occurrence of *Andricus *and *Cynips *gallwasp hosts for *Megastigmus stigmatizans *within the Western Palaearctic.

host code	host species	Iberia	central Europe	eastern Mediterranean	northern Europe
1	*A. caputmedusae*		yes	yes	
2	*A. coriarius*	yes	yes	yes	
3	*A. coronatus*		yes	yes	
4	*A. curtisii*		yes	yes	
5	*A. dentimitratus*	yes	yes	yes	
6	*A. grossulariae*	yes	yes	yes	invader
7	*A. hungaricus*		yes		
8	*A. infectorius*		yes	yes	
9	*A. kollari*	yes	yes	yes	invader
10	*A. lucidus*		yes		invader
11	*A. megalucidus*			yes	
12	*A. pictus*	yes			
13	*A. quercuscalicis*		yes		invader
14	*A. quercustozae*	yes	yes		invader
15	*A. sadeghii*			yes	
16	*A. sternlichti*		yes	yes	
17	*C. longiventris*		yes	yes	native
18	*C. quercusfolii*		yes	yes	native

All galls attacked are induced on *Quercus *section Quercus oaks by asexual generations. Host codes are used for hosts in Additional file 1.

## Methods

### Sample collection

We reared 247 *M. stigmatizans *specimens from host galls collected throughout the Western Palaearctic and stored emerged adults at -20°C in 100% ethanol. At least 20 individuals were sampled from a range of hosts in each of 10 major regions relevant to our three hypotheses: Iran, Lebanon, Turkey, the northern Balkans (Hungary and eastern Austria), Italy, Germany/central France, western France, Spain/Portugal, Morocco, and Britain (Figure 1, Additional file 1). Smaller numbers were obtained from the southern Balkans (Greece and Bulgaria). Our analysis incorporated 29 previously sampled individuals from Iberia and western France [19] and 17 additional samples from this region. Only one *M. stigmatizans *individual was sampled from each gall to minimise any impact of sampling siblings on population genetic analyses.

### Molecular methods

Sequencing procedures followed Nicholls *et al*.[22]. A 590 base pair fragment of the mitochondrial cytochrome *b *gene (cyt *b*) was amplified for all samples using the primers CP1 and CP2 [23]. This fragment gave a total of 55 haplotypes, with 47 variable positions (22 parsimony informative). To increase signal from the mitochondrial genome, one individual from all but four of the cyt *b *haplotypes was also sequenced for the Folmer barcode region of the cytochrome *c *oxidase subunit I gene (COI; 652 base pairs) using the primers LCO and HCO [24]. This added a further 23 variable characters, 14 of which were informative. One individual of the congeneric oak gall parasitoids *M. dorsalis *and *M. synophri *was sequenced for both genes for use as outgroups in the combined gene analysis. Sequences are available from GenBank, accession numbers FJ026622-FJ026729.

To increase resolution of genetic structure and to corroborate the independent evolutionary status of mitochondrial lineages [25], we genotyped 227 of the 247 individuals for 13 nuclear microsatellite loci (Mst2, Mst3, Mst4, Mst6, Mst9, Mst11, Mst12, Mst13, Mst14, Mdo1A, Mdo6, Mdo7 and Mdo11) following protocols in Garnier *et al*.[26]. PCR fragments were sized on an ABI 3730 capillary machine and scored using ABI's custom software GeneMapper v4.0.

### Phylogenetic inference from mitochondrial DNA

Basecalling of sequences was confirmed using Sequence Navigator[27]. Sequences were checked for an open reading frame and aligned using DNAstar (DNAstar Inc., Madison WI, USA). Phylogenetic relationships among unique *M. stigmatizans *cyt *b *haplotypes were reconstructed using MrBayes v3.1.2 [28]. The data were partitioned by codon position, with substitution rates allowed to vary among partitions. Since some categories of transversions were not present within each partition the data were initially modelled using a HKY+I+G model for each codon position. This model was then simplified following the procedure in Nicholls *et al*.[22], resulting in HKY+I+G, HKY and HKY+I for first, second and third positions respectively. MrBayes analyses incorporated two independent runs of 6,000,000 generations, each with 4 chains and a temperature of 0.15. Parameters and trees were sampled every 1000 generations, with the final 500,000 generations used for assessing tree topology and node support. Convergence of parameters between runs was assessed using Tracer v1.4 [29].

For the combined cyt *b *and COI data, partitioning by both gene and codon position produced similar parameter estimates for corresponding codon positions and the same tree topology as partitioning by codon position only, so final analyses used this simpler three partition model. Application of the procedure described above for cyt *b *resulted in adoption of HKY+I as the model for each codon position, with a constant clock-like mutation rate.

### Analyses of microsatellite data

Individuals were screened for 13 loci (see Additional file 1) with 3 to 32 (mean = 13.9) alleles per locus. As wasps are haplodiploid, with haploid males and diploid females, only diploid (i.e. female) genotypes were initially used to assess concordance with the assumptions of further population genetic analyses. We tested for departures from Hardy-Weinberg and linkage equilibrium using Arlequin v2.001 [30] within each of the sampling regions specified above. Significance levels were adjusted for multiple tests using a sequential Bonferroni correction and a table-wide alpha of 0.05. Significant departures were obtained for 23 of 113 tests of Hardy-Weinberg equilibrium and 16 of 6259 tests of linkage equilibrium; however, these departures showed no consistency across loci or populations so all data were included in subsequent analyses.

Phylogeographic structure in the complete microsatellite data was assessed using Structure[31] to identify the number (*K*) of discrete population clusters in the data, and then mapping the geographic extent of these clusters. In these analyses, the second (non-existent) allele in males was coded as missing to allow inclusion of both male haplotypes and female genotypes in one analysis; separate analyses using only the male or female datasets gave almost identical results (data not shown). Analyses incorporating *K *values of 1-16 were run for 1,000,000 generations with a burn-in of 100,000 generations, with convergence in estimated parameter values checked over 10 independent runs for each *K*. An admixture model was used, allowing individuals to have ancestry from multiple populations, with population allele frequencies assumed to be independent.

Relationships among the clusters found by Structure were assessed using only those individuals with limited evidence of admixture (a minimum of 80% of their genotype assigned to a single population). Nei's genetic distance [32] was calculated between all pairwise combinations of clusters and then used to construct a neighbour-joining tree in PHYLIP[33]. Node support was assessed from 1000 bootstrap replicates based on resampled data matrices of loci across clusters. A second metric based on pairwise F_ST _between clusters was also calculated, and gave almost identical results (data not shown).

## Results

### Regionally diagnostic genetic structure in *M. stigmatizans' *native range

**Figure 2:**
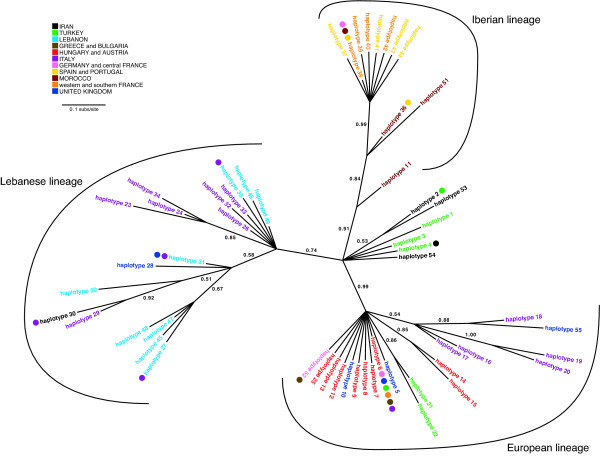
**Unrooted Bayesian 50% majority-rule consensus phylogram for 55 *Megastigmus stigmatizans *cytochrome *b *haplotypes**. Haplotypes (and dots after haplotype names) are coloured by the geographic region of occurrence. Haplotypes are numbered as in Additional file 1. Major lineages mentioned in the text are labelled. Numbers next to branches are Bayesian posterior probabilities.

**Figure 3:**
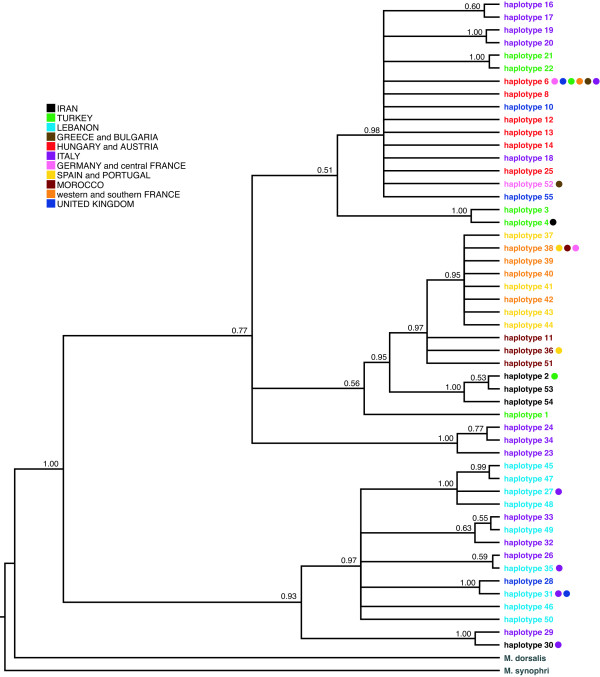
**MrBayes 50% majority-rule consensus tree for the combined cyt *b*/COI genes from *Megastigmus stigmatizans***. Terminal taxa are labelled using cyt *b *haplotype numbers as in Figure 2 and Additional file 1, and are coloured by geographic occurrence. Numbers next to branches are Bayesian posterior probabilities.

**Figure 4:**
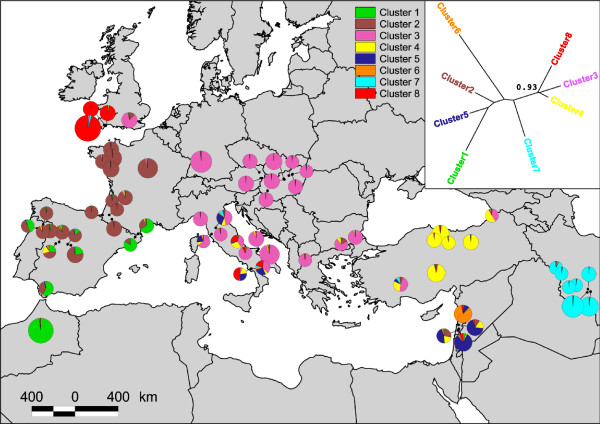
**Distribution of the eight clusters identified in the Structure analysis of *Megastigmus stigmatizans *multi-locus genotypes**. Data are presented as the proportion of the sample at each site that was assigned to each cluster; size of circles corresponds to the number of individuals at that site (n = 1-23). The insert shows the relationships among clusters with bootstrap support, determined by neighbour-joining analysis of Nei's genetic distance.

Genetic variation in both mitochondrial and microsatellite datasets was diagnostic of major regions across the native range of *M*. *stigmatizans*, with strong concordance between an individual's mtDNA lineage and the cluster to which the majority of its microsatellite genotype was assigned (χ^2 ^= 449.8, d.f. = 21, *P *≈ 0). Three well-supported regional clades were resolved in the cyt *b *(Figure 2; named the European, Iberian and Lebanese lineages) and combined mitochondrial (Figure 3) datasets, with multiple Turkish and Iranian haplotypes distinct from the major lineages. The microsatellite data provided greater resolution, with strongest support for eight population clusters (posterior probability of ~1 for *K *= 8) showing similar geographic structuring to the mitochondrial data (Figure 4). Few individuals showed significant evidence of admixture (microsatellite alleles originating from different clusters; see Additional file 2), suggesting limited migration between these spatially separated populations. Balkan populations were characterised by cyt *b *haplotypes in the European lineage and microsatellite genotypes in cluster 3. Iberian populations were characterised by cyt *b *haplotypes in the Iberian lineage and microsatellite genotypes in clusters 1 or 2. Eastern populations were the most genetically diverse for both marker types, and were characterised by cyt *b *haplotypes either in the Lebanese lineage (Lebanon, Iran) or in a distinct central polytomy (Turkey, Iran), with microsatellite genotypes in clusters 4-8. This regionally-diagnostic variation allowed unequivocal inference of the origin of invading *M. stigmatizans *populations across their non-native range.

### Origins of non-native *M. stigmatizans *in northern Europe

The molecular data show that all three hypothesised mechanisms have contributed to range expansion in *M. stigmatizans*. The **Host Pursuit Hypothesis **is supported by assignment of cyt *b *sequences from Britain, France and Germany to the European lineage (Figure 2), and the observation that European microsatellite genotypes dominate populations in Germany and south-eastern Britain (cluster 3 in Figure 4). While Italy could potentially have been the source of these European genotypes, none of the other microsatellite clusters that are present at high frequency in Italy (clusters 4, 5 and 8) occurred further north in continental Europe, indicating that these northern *M. stigmatizans *populations are derived from the remainder of the refugial distribution of cluster 3 in the Balkans, as is the case with their hosts. Notably, European lineage mitochondrial haplotypes were more widespread than microsatellite genotypes from the same origin, occurring through all but southern-most France and throughout southern Britain. Genetic diversity within the European mitochondrial lineage declined with distance from the Balkan origin (only five European lineage cyt *b *haplotypes were observed in northern Europe compared to 10 in the Balkan peninsula), and haplotype 6 was particularly widespread and abundant (found in 54% of European lineage individuals and occurring throughout the lineage's geographic range).

The **Host Shift Hypothesis **was supported by the presence of Iberian lineage cyt *b *haplotypes in central and northern France (Figure 2), and widespread occurrence of cluster 2 microsatellite genotypes across France into south-eastern Britain (site 70; Figure 4). As with the European mitochondrial lineage, northern populations showed reduced diversity with haplotype 38 dominant; however, some individuals sampled from central France, northern France and Britain that were assigned to the Iberian microsatellite cluster 2 had European lineage mitochondrial haplotypes (see Additional file 2).

The **Introduction Hypothesis **was supported for both Britain and Italy by inclusion of cyt *b *haplotypes from these regions in the Lebanese lineage (Figure 2). Cluster 8 in the microsatellite data also showed the same disjunct distribution, at high frequency in Britain and some Italian populations, and low but significant frequency in some Middle Eastern populations (Figure 4; Additional file 2). Neither the mitochondrial Lebanese clade nor the microsatellite cluster 8 occurred in geographically intervening regions, thus excluding the possibility that any natural range expansion event could have given rise to this pattern. The dominance of microsatellite cluster 8 in south-western Britain is striking, with all but one microsatellite genotype from sites 67-69 allocated to this cluster; the final genotype showed admixture with another eastern population, the Iranian cluster 7. Equally striking is the lack of nuclear admixture between south-western British populations and the microsatellite genotypes found in south-eastern Britain and neighbouring continental Europe; however, European lineage mitochondrial haplotypes were sampled from south-western individuals (see Additional file 2). These patterns strongly support the Introduction Hypothesis for British *M. stigmatizans*, with no subsequent cross-Channel genetic exchange with mainland Europe other than in the south-east.

**Figure 5:**
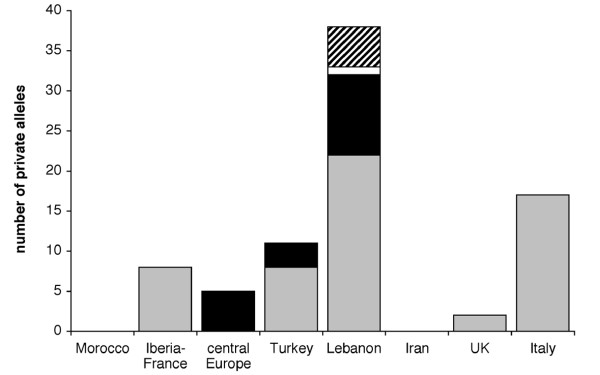
**Number of private alleles in per geographic region for 13 *Megastigmus stigmatizans *microsatellite loci**. Grey bars indicate alleles private to a single region, alleles shared only with Italy are in black, alleles shared with both Italy and Britain are in white, and alleles shared only with Britain are indicated by diagonal lines.

The Introduction Hypothesis also explained microsatellite genotype patterns seen in Italy. These populations are genetically diverse, with contributions from central Europe (cluster 3) and multiple eastern populations (clusters 4, 5, 7 and 8). However, in contrast to British populations, more than a third of Italian individuals showed a strong signature of genetic admixture between these sources (see Additional file 2). The distribution of alleles shared by Britain or Italy and other populations underlines support for the Introduction Hypothesis. Nineteen alleles were shared only by Britain or Italy and either Lebanon or Turkey (Figure 5). Other than five alleles shared between Italy and central Europe (a pattern expected given the evidence grouping these populations) no other region shared alleles exclusively with Britain or Italy, supporting a process of introduction from these eastern source populations.

## Discussion

### The complex invasion history of *M. stigmatizans*

Our results reveal a complex invasion history for *M. stigmatizans *in northern Europe, with strong support for all three invasion hypotheses. Some individuals are clearly derived from the Balkan native range of both their gallwasp hosts and *Q. cerris*, the oak upon which the entire trophic cascade depends [9,10,34], supporting the **Host Pursuit Hypothesis**. Unlike some invading host gallwasps [10,34] but in common with the gallwasp *A. kollari *[9], Balkan parasitoid populations have colonised only as far as south-eastern Britain. In contrast to Iberian host-alternating gallwasps, Iberian *M. stigmatizans *have expanded their distribution northwards, dominating invading populations throughout France and supporting the **Host Shift Hypothesis**. Our data confirm preliminary haplotype-based observation of this pattern [19] and extend the limits of range expansion by Iberian populations to south-eastern Britain (microsatellite cluster 2).

Finally, most British populations and some Italian individuals show evidence of eastern ancestry consistent with the **Introduction Hypothesis**. This is consistent with the known import of host galls to Britain during the 1840s [9,35]. Eastern genotypes in Italy probably derive from much earlier trade, perhaps dating back to Roman times [9]. Genetic data for British *A. kollari *suggested introduction from two distinct sources, neither of which matched sampled populations [9]. A similar pattern applies to *M. stigmatizans*; although some British and Lebanese cyt *b *sequences indicate a close relationship, none of the sampled eastern populations share the dominance of microsatellite cluster 8 characteristic of south-western Britain, and clear structure in the microsatellite data argue against the cluster 8 source population being in southern (cluster 5) or northern (cluster 6) Lebanon. One possibility is that the dominance of cluster 8 in Britain results from a genetic bottleneck in the introduced population [10]. However, the existence of a parallel pattern with no obvious loss of genetic variation in introduced *A. kollari *suggests the presence, at least in the 1840s, of a genetically discrete oak gall community in an eastern Mediterranean region that we have yet to sample. Neighbour-joining analysis grouped cluster 8 with the Turkish cluster 4, so this unknown source population may have been north of Lebanon, perhaps in Syria or south-eastern Turkey as was also suggested for *A. kollari *and consistent with Aleppo, in Syria, being a probable centre of the oak gall trade [9]. Although this putative origin could potentially be located by more sampling, extensive deforestation in this region since the mid-nineteenth century [36] may mean that the introduced eastern genotypes of both host and parasitoid that dominate British populations no longer exist at their geographic source.

### Modes of range expansion in *M. stigmatizans*

Our data show that persistent patterns have established in the nuclear genetic structure of non-native *M. stigmatizans *populations. In southwestern Britain, eastern genotypes have persisted for 170 years with no evidence of significant cross-Channel gene flow from western France. On mainland Europe, both Balkan and Iberian populations have spread without known human assistance over at least 1000 km of previously unoccupied habitat over at most 400 years, with little evidence of mixing except where both contribute genetic diversity to the extreme southeast of Britain. The persistent integrity of eastern genotypes in Britain and evidence of limited cross-channel gene flow parallels exactly the pattern seen in *A. kollari *[9]. Other gallwasp invasions indicate that these insects can cross the Channel without significant loss of genetic diversity [10], so lack of dispersal is unlikely to explain the observed pattern. Instead, the first arrivals may have established a genetic dominance in populations that was resistant to rare arrivals from genetically divergent populations, as predicted by leptokurtic dispersal models [37,38]. Such genetic dominance can establish very quickly [10], so rapid establishment of host-shifting colonists from Iberia could also explain why central European genetic diversity in *M. stigmatizans *is conspicuously absent from France. This contrasts with extensive genetic admixture between multiple eastern and European populations in Italy which is consistent with co-existence over much longer timescales, a pattern mirrored in refugial Spain and Lebanon where multiple microsatellite clusters have a long history of co-existence.

However, patterns in the mitochondrial genome differ subtly from those described above for the nuclear genome. Introgression of central European haplotypes into both Iberian and eastern nuclear backgrounds was seen in France and Britain, respectively. This perhaps indicates a degree of uni-directional female-biased dispersal followed by backcrossing with local populations, or alternatively introgression of selectively advantageous Balkan mitochondria. Either way, it highlights the inability of populations invading from multiple sources to maintain complete genetic distinctiveness. So although genetic diversity within specific source lineages decreased as populations expanded northwards [see also [10]], total genetic diversity at the limits of the non-native range remained high due to contributions from multiple source lineages. This parallels patterns seen in other taxa expanding their distributions, either naturally or with human assistance, across Europe from multiple sources [9,39].

### Host shifts and the evolution of oak gall communities

The ability of Iberian *M. stigmatizans *to colonise France highlights the fact that herbivores and their natural enemies may face very different constraints on range expansion. While Iberian gallwasps have failed to shift from *Q. suber *in Iberia to *Q. cerris *north of the Pyrenees, *M. stigmatizans *was able to shift from Iberian hosts to invading hosts once these came within reach. Unlike other species [40], no discernable population bottleneck accompanied this shift, suggesting the invaders were easy to exploit as hosts. Ability to exploit unfamiliar hosts is also supported by the genetic data in Britain. Eastern *M. stigmatizans *genotypes must originally have been associated only with co-introduced eastern *A. kollari*. However, our sampling revealed eastern *M. stigmatizans *genotypes in individuals attacking another gallwasp host, *A. quercuscalicis*. This gallwasp invaded Britain from the Balkans in the late 1950s [10] and is absent from the eastern Mediterranean (Table 1). Eastern origin *M. stigmatizans *must thus have shifted host from *A. kollari *to *A. quercuscalicis *in the last 50 years, equating to a maximum of only 50 generations as *M. stigmatizans *is univoltine. Recent additional British sampling has also revealed *M. stigmatizans *attacking a novel native gallwasp host, *Biorhiza pallida *(K. Schönrogge & J.A. Nicholls unpubl. data). These host shifts in France and Britain suggest that oak or gall traits required for host recognition by *M. stigmatizans *are broad enough to accommodate any divergence among host populations in different regions of the Western Palaearctic that have been separated for multiple glacial cycles [19,41,42], implying co-evolutionary interactions such as local adaptation between refugial populations of *M. stigmatizans *and its gallwasp hosts are weak or non-existent. Although the timescale of such divergence is difficult to estimate, the most recent interglacial allowing possible mixing of oak communities between Iberia and central Europe was around 130,000 years ago [43]. However, both the mitochondrial tree (Figure 3) and the microsatellite data (insert in Figure 4) indicate that Iberian and central European populations have a non-sister relationship, suggesting that these populations have had independent evolutionary histories for a much longer period on the same timescale (or longer than) the divergence between European and Middle Eastern populations. It has been argued that the massive range shifts caused by Pleistocene glaciations [44,45] may have selected against highly co-evolved species associations in favour of a degree of generalist opportunism [46]. This may explain why most oak gall parasitoids exploit suites of hosts sharing similar gall phenotypes [47]. Thus patterns in *M. stigmatizans*, and in oak galls more generally, support a major and continuing role for ecological sorting in the recent assembly of these communities.

While *M. stigmatizans *and its gallwasp hosts have differing invasion histories in northern Europe, they share similar patterns of genetic diversification across their native ranges. The major divisions between *M. stigmatizans *lineages in Iberia, central Europe and the Middle East correspond to major Pleistocene refugia for many Western Palaearctic taxa [44,45]. The placement of Iranian and Turkish sequences in a central unresolved polytomy in the cyt *b *tree (Figure 2) and the basal placement of the Lebanese lineage in the combined mitochondrial analysis (Figure 3) also support an overall eastern origin for *M. stigmatizans*. These patterns are shared with other oak gallwasp and parasitoid species examined to date [9,12,22,41,48,49] supporting hypotheses of an eastern origin for Western Palaearctic oak gallwasp communities as a whole [7].

### Implications for the release of biocontrol agents

Oak gallwasp communities illustrate several major issues associated with impacts of introduced species on native communities. Substantial changes in community structure can develop rapidly [11,13,14,20], and parasitoids associated with invading hosts can reach very high population densities, with potential impacts on native hosts through apparent competition [11,13,50]. In addition, limitations to range expansion experienced by their hosts are irrelevant to parasitoids as long as suitable alternative hosts exist. This significance of biotic interactions on species distributions has been described in other systems, and underlines the risk of applying climate-envelope approaches to modelling community level responses to perturbation [3,51,52]. *Megastigmus stigmatizans *shows that understanding range expansion in parasitoids, and predicting impacts in associated communities, requires identification of the factors defining their host choice, and hence of the potential for range expansion via host shifts. This issue is of immediate relevance in Europe, where the biocontrol agent *Torymus sinensis *has been released to control the invading chestnut gallwasp *Dryocosmus kuriphilus*. Although *T. sinensis *is an effective biocontrol agent previously deployed in Asia and America [20,21], its ability to attack non-target hosts remains largely untested. Chestnut gallwasps are recognised as hosts by native oak gall parasitoids [20], so *T. sinensis *may likewise attack native oak gallwasps. If so, the potential exists for it not only to pursue its invading pest host, but also (as *M. stigmatizans *has done) to shift to non-target hosts and disperse much more widely in the Western Palaearctic. Until this parasitoid's ability to exploit non-target hosts is understood, the wider impacts of its ongoing release remain impossible to predict.

## Conclusions

In conclusion, this study highlights the major role for ecological sorting processes in the recent assembly of complex communities in response to anthropogenic disturbance. Different trophic levels may face very different ecological constraints on range expansion, resulting in varying phylogeographic histories of species interacting within the same community. The multitude of origins of invading natural enemy populations in this study emphasises the diversity of mechanisms requiring consideration when trying to predict the consequences of human-mediated ecological perturbations such as biological invasions or the intentional release of biological control agents.

## Authors' contributions

JAN helped design the study, generated much of the mitochondrial data, performed the statistical analyses and wrote the manuscript. PFU generated and analysed the microsatellite data. AH initiated the sampling and generated some mitochondrial data. GM, GC, JLNA, JPV and MT provided extensive assistance with sample collection. GNS and KS assisted with study design and conception, and writing of the manuscript. All authors read and approved the final manuscript.

## Supplementary Material

Additional file 1 **Collection details and genetic data for each sampled *Megastigmus stigmatizans *individual**. Excel spreadsheet containing sampling location and host gall for each specimen used, along with nuclear genotypes assessed over 13 microsatellite loci (scored as the allele size in base pairs, missing values coded as 0) and cytochrome *b *haplotype. Site numbers correspond to those in Figure 1. Females are diploid so have two alleles at each microsatellite locus, males are haploid so have one allele. An asterisk next to the cyt *b *haplotype number indicates that individual was also sequenced for the cytochrome oxidase I fragment. Host gall code corresponds to species listed in Table 1. Click here for file

Additional file 2 **Estimated membership coefficients of the eight clusters derived from the Structure analysis for each *Megastigmus stigmatizans *individual**. Excel spreadsheet containing output of the Structure analysis, with the highest membership scores (summing to >80% membership) highlighted in bold. The final column indicates mitochondrial lineage membership. Click here for file
